# Thymoquinone synergizes gemcitabine anti-breast cancer activity via modulating its apoptotic and autophagic activities

**DOI:** 10.1038/s41598-018-30046-z

**Published:** 2018-08-03

**Authors:** Hanan A. Bashmail, Aliaa A. Alamoudi, Abdulwahab Noorwali, Gehan A. Hegazy, Ghada AJabnoor, Hani Choudhry, Ahmed M. Al-Abd

**Affiliations:** 1 Departement of Clinical Biochemistry, Faculty of Medicine, King Abdulaziz University, Jeddah, Saudi Arabia; 2 Stem Cell Research Unit, King Fahad Medical Research Center, Jeddah, Saudi Arabia; 3 Department of Hormones, Medical Division, National Research Centre, Giza, Egypt; 4 Department of Biochemistry, Faculty of Science, King Abdulaziz University, Jeddah, Saudi Arabia; 5 Department of Pharmacology, Medical Division, National Research Centre, Giza, Egypt; 6 Biomedical Research Section, Nawah Scientific, Mokkatam, Cairo, Egypt; 7 Depaertment of Pharmaceutical Sciences, College of Pharmacy, Gulf Medical University, Ajman, United Arab Emirates

## Abstract

The use of anti-cancer adjuvant therapy is rationalized by potentiating the efficacy, and/or protecting from major side effects of chemotherapeutics. Thymoquinone (TQ) is a naturally occurring compound with cumulative evidence of anti-cancer properties. In this study, we assessed the chemomodulatory potential of TQ to gemcitabine (GCB) against human breast adenocarcinoma (MCF-7), and ductal carcinoma (T47D) cells. TQ showed cytotoxic effects against MCF-7 and T47D with IC_50_’s of 64.9 ± 14 µM and 165 ± 2 µM, respectively. The IC_50_’s of GCB against MCF-7 and T47D were 0.9 ± 0.18 µM and 14.3 ± 2.8 µM and were significantly reduced after combination with TQ to 0.058 ± 12 µM and 2.3 ± 0.2 µM, respectively. The CI- values were indicative of synergism in MCF-7 and T47D cells (0.15 and 0.30, respectively). Further investigation showed that GCB caused significant anti-proliferative effect reflected by increasing cell population in S-phase in both cell lines. TQ potentiated GCB-induced anti-proliferative activity in both cell lines. GCB induced considerable apoptosis in T47D cell line, and TQ significantly increased GCB-induced apoptotic effects by 1.5 to 3.6 folds. Interestingly, GCB, TQ and their combination induced significant autophagic cell death in the apoptosis defected MCF-7 cells. In addition, TQ, GCB and their combination depleted breast cancer associated stem cell (CD44^(+)^/CD24^(−)/(low)^) clone within MCF-7 and T47D cells by 3.8% to 27.5%. In conclusion, TQ showed promising chemomodulatory effects to GCB against breast cancer cells via inducing apoptosis, necrosis and autophagy, in addition to depleting tumor associated resistant stem cell fraction.

## Introduction

Cancer is a global health problem which is increasing with population growth, aging, and inappropriate lifestyle^1^. Breast cancer is the most common type of cancer in females and there are over one million newly diagnosed breast cancer cases, and 502,000 breast cancer related deaths per year^2^. Breast cancer tissue is made up of different cell types expressing different cell surface markers, with different microscopic appearances and growth rates^3^. Breast cancer stem cells (BCSC) are depot cell clone characterized by indefinite self-renewal ability, and high resistance to chemotherapy^4^. Various breast cancer treatment options such as; surgery, radiation, chemotherapy, hormonal and targeted therapy are currently in clinical practice^5^. However, targeting and depleting the intratumoral associated cancer stem cells remain to be clinical as well as scientific challenge.

Gemcitabine (GCB) is a nucleoside analog chemotherapy which is widely used for different types of neoplasia and was clinically approved for the treatment of metastatic breast cancer since 2004^6^. It requires triphosphate activation to get incorporated into DNA double helix resulting in inhibition of DNA synthesis^7^. Despite the widespread use of GCB, it suffers from many drawbacks such as; lack of selectivity, exaggerated normal tissue toxicity, and most importantly emergence of tumor resistance^6,8^. Resistance to GCB treatment might appear in the form of tumor relapse/recurrence and remote organ metastasis^9^.

Natural compounds and even crude medicinal plants are believed to be promising source of alternative anti-cancer remedy. They are well-known to suppress or block the carcinogenic processes^10^. Amongst, *Nigella sativa* is extensively studied for potential anticancer properties. It was even described as a miracle herb since many studies revealed its outstanding pharmacological potential^11^. Thymoquinone (TQ) is one of the major bioactive compounds isolated from *Nigella sativa*, it possesses anti-inflammation, anti-hypertensive, anti-oxidant, and anti-cancer effects^12,13^. Combination of natural compounds with conventional cancer chemotherapy showed promising outcomes and gained the attention of scientists worldwide due to enhancing the anti-cancer efficacy without increasing normal tissue toxicity^14^ In our previous work, combination of TQ with cisplatin diminished the resistance fraction to cisplatin and improved its anticancer activity against head and neck cancer cells^15^. Yet, TQ might overcome resistance to GCB and would be a potential successful combination therapy for breast cancer. Therefore, the aim of the current work is to investigate the potential chemomodulatory effects of TQ to the cytotoxic profile of GCB against human breast cancer cell lines.

## Results

### TQ improves the cytotoxicity of GCB in breast cancer cell lines

To study the influence of TQ on the cytotoxic profile of GCB against breast cancer cells, dose response curves of GCB, TQ and their combination were conducted against MCF-7 and T47D cells (Table 1).

**Table 1:** Combination analysis for the cytotoxicity of TQ and GCB against MCF-7 and T47D breast cancer cell lines.

Exposure time	MCF-7		T47D	
24 h	IC_50_ (µM)	R-value (%)	IC_50_ (µM)	R-value (%)
GCB	32.6 ± 3.6	2.7 ± 0.6	7.6 ± 0.7	6.5 ± 0.5
TQ	80.1 ± 9.8	55.5 ± 1.2	>100	N/A
GCB + TQ	3.4 ± 1.3	0.1 ± 0.03	0.3 ± 0.02	4.3 ± 0.2
**CI-value**	**Synergism/0**.**53**		**Synergism/less than 0**.**34**	
**48** **h**				
GCB	32.2 ± 1.1	0.5 ± 0.1	6.4 ± 1.4	2.3 ± 0.5
TQ	32.7 ± 1.1	1.2 ± 0.5	104 ± 3.7	N/A
GCB + TQ	3.6 ± 0.7	0.9 ± 0.1	0.27 ± 0.07	1.8 ± 0.4
**CI-value**	**Synergism/0**.**22**		**Synergism/0**.**1**	
**72** **h**				
GCB	0.9 ± 0.18	0	14.3 ± 2.8	3.2 ± 4.5
TQ	64.9 ± 14.5	1.6 ± 1.3	165.1 ± 2.8	0. 1 ± 0.15
GCB + TQ	0.06 ± 0.01	0	2.3 ± 0.2	0
**CI-value**	**Synergism/0**.**15**		**Synergism/0**.**25**	

Data is presented as mean ± SD; n = 3.

**Figure 1:**
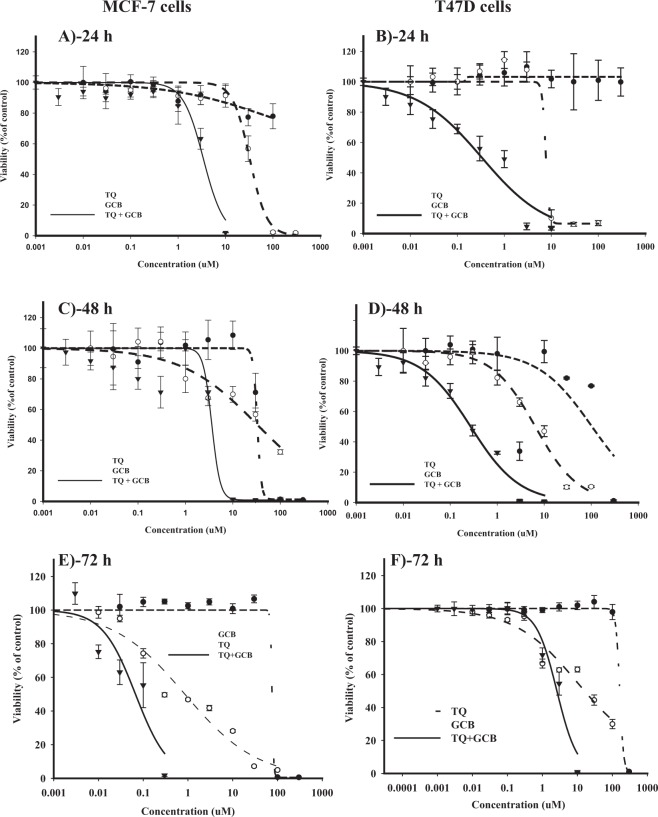
The chemomodulatory effect of TQ on the cytotoxicity of GCB in MCF-7 (**A**,**C** and **E**) and T47D (**B**,**D** and **F**) breast cancer cell lines. Cells were exposed to serial dilution of GCB, TQ or their combination for 24 h (**A** and **B**), 48 h (**C** and **D**) 72 h (**E** and **F**). Cell viability was determined using SRB-assay and data are expressed as mean ± SD (n = 3).

In MCF-7 cells, exposure to GCB for 24 h and 48 h did not induce considerable cytotoxicity (IC_50_’s were 32.6 ± 3.6 and 32.2 ± 1.1, respectively) (Fig. 1A and C). Further exposure of MCF-7 cells to GCB (72 h) exerted gradient cytotoxic activity with increasing concentration; viability started to drop significantly at concentration of 0.3 μM with IC_50_ of 0.9 ± 0.18 μM (Fig. 1E). TQ exerted weak cytotoxic profile against MCF-7 cells after treatment for 24 h, 48 and 72 h (IC_50_’s were 80.1 ± 9.8, 32.7 ± 1.1 and 64.9 ± 14.5, respectively). TQ-induced cytotoxic activity started after 10 μM concentration. Further exposure to higher concentration of TQ induced sudden viability drop at 100 μM (Fig. 1A,C and E). Equitoxic combination of TQ with GCB significantly improved the cytotoxic profile of GCB decreasing its IC_50_ by 9.5 to 15.5 folds (3.4 ± 1.3, 3.6 ± 0.7 and 0.06 ± 0.01 µM after 24 h, 48 h and 72 h exposure, respectively). The calculated CI- values ranged from 0.15 to 0.53, which are indicative of strong synergism at all exposure time points (Table 1).

In T47D cell line, GCB exerted gradient cytotoxic activity with increasing concentration at all exposure time points; viability started to drop at concentrations equal to or higher than 1 μM with an IC_50_’s of 7.6 ± 0.7, 6.4 ± 1.4 and 14.3 ± 2.8 μM after 24 h, 48 h and 72 h, respectively. TQ treatment showed sudden cytotoxic effect at concentrations higher than 100 μM; IC_50_ of TQ was found to be higher than 100 μM at all exposure time points (Fig. 1B,D and F). Equitoxic combination of TQ with GCB significantly improved the cytotoxic profile of GCB, inducing remarkable decrease in its IC_50_ by 6.2 to 25 folds (0.3 ± 0.02, 0.27 ± 0.07 and 2.3 ± 0.2 µM). Combination analysis revealed a strong synergism between GCB and TQ with CI-values ranging from 0.1 to 0.34 (Table 1).

### Effect of GCB, TQ and their combination on cell cycle distribution of breast cancer cells

**Figure 2:**
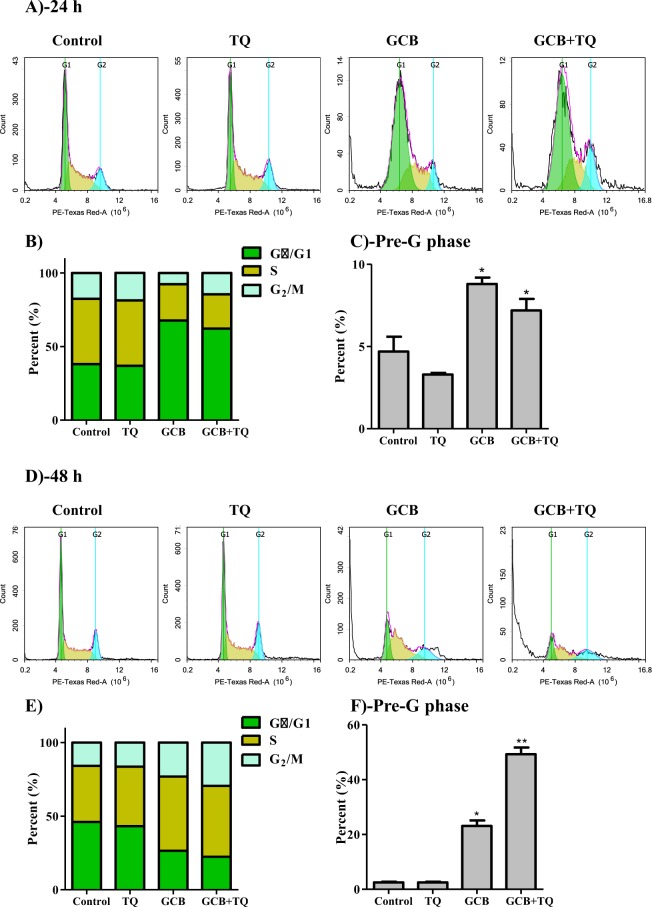
Effect of GCB, TQ and their combination on the cell cycle distribution of MCF-7 cells. Cells were exposed to GCB, TQ or their combination for 24 h (**A**–**C**) or 48 h (**D**–**F**). Cell cycle distribution was determined using DNA cytometry analysis and different cell phases were plotted (**B**,**E**) as percentage of total events. Sub-G cell population was plotted as percent of total events (**C**,**F**). Data is presented as mean ± SD; n = 3. *Significantly different from control group. **Significantly different from GCB treatment.

To assess the influence of GCB, TQ and their combination on the cell cycle distribution of breast cancer cells, MCF-7 and T47D cells were treated with the pre-determined IC_50_’s of treatments under investigation for 24 and 48 h, and assessed for DNA content using flow cytometry. In MCF-7, TQ alone did not induce any significant change in all cell cycle phases. However, GCB caused significant anti-proliferative effect manifested by increasing the cell population at G_0_/G_1_ phase after 24 h from 38.1 ± 3% to 67.7 ± 1.2% (Supp. Table 1). Further exposure (48 h) of MCF-7 to GCB induced significant S-phase arrest increasing its cell population from 38.0 ± 1.9% to 50.4 ± 3.2%. Combining TQ with GCB did not further increase GCB-induced antiproliferative effects either in G_0_/G_1_-phase or S-phase (Fig. 2A,B,D,E). After 24 h, GCB alone or GCB in combination with TQ induced significant increase in pre-G cell population from 4.7 ± 1.5% to 8.8 ± 0.6% and 7.2 ± 1.2%, respectively. Longer exposure (48 h) of MCF-7 cells to GCB significantly induced more cell death manifested by 23.1 ± 3.4% of cells in the pre-G phase compared to untreated cells (2.5 ± 0.6%). Further exposure of MCF-7 cells to combination of GCB with TQ for 48 h resulted in significantly higher cell death; pre-G cell population increased to 49.3 ± 4.2% compared to GCB treatment alone (Fig. 2C,F) and (Supp. Table 2).

**Figure 3:**
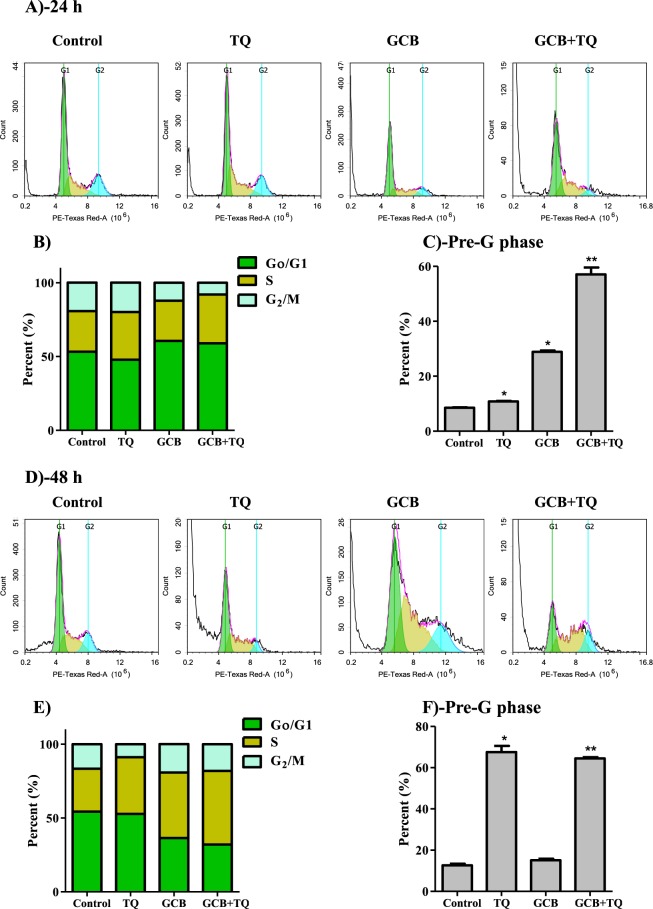
Effect of GCB, TQ and their combination on the cell cycle distribution of T47D cells. Cells were exposed to GCB, TQ or their combination for 24 h (**A**–**C**) or 48 h (**D**–**F**). Cell cycle distribution was determined using DNA cytometry analysis and different cell phases were plotted (**B**,**E**) as percentage of total events. Sub-G cell population was plotted as percent of total events (**C**,**F**). Data is presented as mean ± SD; n = 3. Data is presented as mean ± SD; n = 3. *Significantly different from control group. **Significantly different from GCB treatment.

In T47D, TQ alone did not cause any significant change in cell cycle distribution after 24 h. However, it induced cell cycle arrest at S-phase after 48 h; cell population within S-phase was increased from 29.1 ± 1.7% to 38.4 ± 0.2%. In addition, GCB induced significant anti-proliferative effect manifested by increasing cells at G_0_/G_1_ phase after 24 h from 53.2 ± 0.6% to 60.5 ± 1.8%. After 48 h, GCB induced significant S-phase arrest increasing its cell population from 29.1 ± 2.7% to 36.5 ± 3.4%. The combination of TQ with GCB for 48 h increased GCB-induced antiproliferative effects manifested by increasing cells in S-phase from 44.3 ± 2.5% to 49.9 ± 1.3% (Fig. 3A,B,D,E) and (Supp. Table 3). After 24 h of treatment, TQ induced significant increase in the pre-G cell population from 8.5 ± 0.3% to 10.8 ± 0.2%. Further exposure (48 h) of T47D to TQ significantly induced more cell death manifested by increased pre-G phase cell population from 12.6 ± 1.4% to 67.6 ± 5.2%. Moreover, GCB alone or GCB in combination with TQ induced significant increase in pre-G cell population from 8.5 ± 0.3% to 28.9 ± 0.9% and 57.1 ± 4.4% after 24 h of treatment, respectively. Longer exposure (48 h) of T47D cells to GCB in combination with TQ resulted in significantly higher cell death compared to GCB treatment alone; cells in pre-G phase was increased from 15.1 ± 1.1% to 64.5 ± 1% (Fig. 3C,F) and (Supp. Table 4).

### Assessment of apoptosis

**Figure 4:**
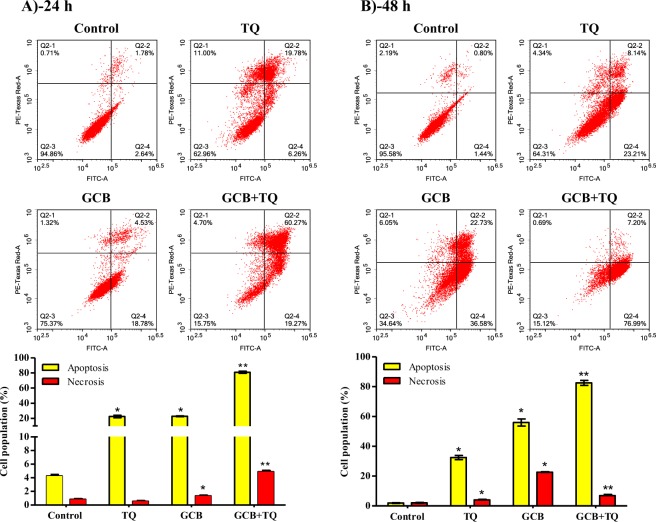
Apoptosis/necrosis assessment in T47D cells after exposure to GCB, TQ and their combination. Cells were exposed to GCB, TQ or their combination for 24 h (**A**) and 48 h (**B**). Cells were stained with annexin V-FITC/PI and different cell populations were plotted as percentage of total events. Data is presented as mean ± SD; n = 3. *Significantly different from control group. **Significantly different from GCB treatment.

To determine the mechanism of cell death (programmed or non-programmed) induced by TQ, GCB and their combination, cells were assessed using Annexin-V/FITC staining coupled with flowcytometry after exposure to the pre-determined IC_50_’s. TQ alone induced significant apoptosis after 24 and 48 h of exposure (22.4 ± 3.1% and 32.4 ± 2.7%, respectively) compared to control untreated cells (4.3 ± 0.4% and 1.9 ± 0.4%, respectively) (Fig. 4). After 24 h, GCB alone or GCB in combination with TQ induced significant apoptotic cell death (22.7 ± 0.9% and 80.9 ± 2.7%, respectively) (Fig. 4A). Prolonged exposure (48 h) of cells to GCB or GCB with TQ significantly induced apoptosis in 55.9 ± 4.7% and 82.5 ± 3.2 of treated cells, respectively (Fig. 4B). In addition, treatment with GCB or GCB combination with TQ for 24 h induced significant necrotic cell death compared to control cell (1.3 ± 0.1%, 4.9 ± 0.2% and 0.84 ± 0.1%, respectively) (Fig. 4A). Prolonged exposure of cells to single treatment of GCB or TQ induced cell necrosis 22.64 ± 0.09% and 4.01 ± 0.3%, respectively. However, TQ in combination with GCB induced necrosis in only 6.9 ± 1.1% of cells (Fig. 4B).

### Assessment of autophagy

**Figure 5:**
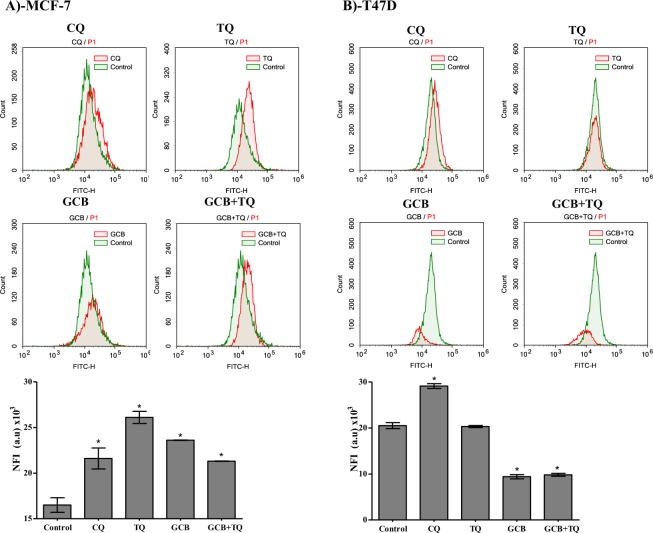
Autophagic cell death assessment in MCF-7 (**A**) and T47D (**B**) cells after exposure to GCB, TQ and their combination. Cells were exposed to GCB, TQ or their combination for 24 h; and were stained with Cyto-ID autophagosome tracker. Net fluorescent intensity (NFI) were plotted and compared to basal fluorescence of control group. Data is presented as mean ± SD; n = 3. *Significantly different from control group. **Significantly different from GCB treatment.

Other than apoptosis, programmed cell death via autophagy represents a big research controversy. Herein, we further investigated the effect of GCB, TQ and their combination on autophagy process within MCF-7 and T47D cells using Cyto-ID autophagy detection dye coupled with flowcytometry. In MCF-7, treatment with GCB or TQ alone increased autophagic cell death by 39.4% and 60.6%, respectively. Combination of TQ with GCB significantly increased autophagic cell death by 29.1% compared to control untreated cells (Fig. 5A).

Unlike MCF-7 cells, TQ alone did not induce any significant change in autophagic cell death in T47D cells. Furthermore, GCB and combination of GCB with TQ significantly decreased autophagic cell death by 54.1% and 52.2%, respectively (Fig. 5B).

### Stem cell detection

**Figure 6:**
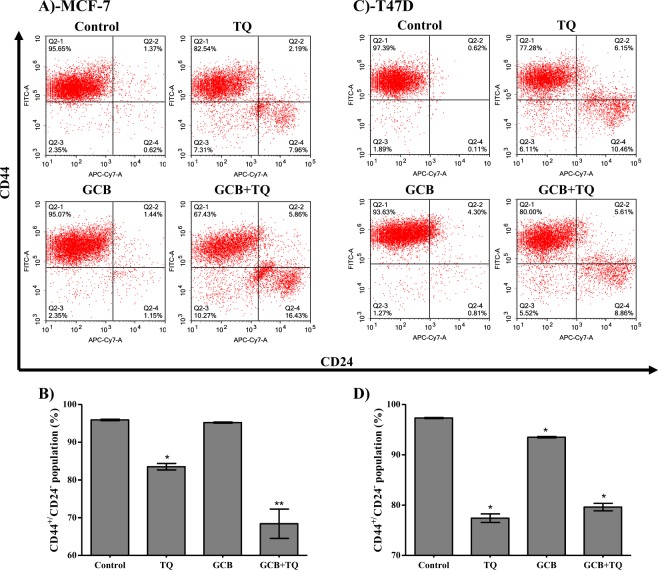
Effect of GCB, TQ and their combination on the expression of CD44 and CD24 stem cell markers. MCF-7 (**A**) and T47D (**B**) cells were exposed to GCB, TQ or their combination for 24 h. Expression levels of CD44 and CD24 were assessed using flow cytometry plotted as percentage of total events. Data is presented as mean ± SD; n = 3. *Significantly different from control group. **Significantly different from GCB treatment.

The effect of GCB, TQ and their combination against tumor associated stem cell clone (CD44^+^/CD24^−^) was assessed using flow cytometry. In MCF-7, TQ alone significantly decreased CD44^+^/CD24^−^ cell clone by 12.4%. However, GCB did not change the percent of CD44^+^/CD24^−^ cell clone. In addition, combination of GCB with TQ significantly decreased CD44^+^/CD24^−^ cells by 27.5% (Fig. 6A and B).

Similarly, TQ alone significantly decreases T47D derived stem cell clone (CD44^+^/CD24^−^) by 19.9% while GCB caused a 3.9% decrease in T47D derived stem cells. Combination of GCB with TQ significantly decreased CD44^+^/CD24^−^ cell clone by 17.7% (Fig. 6C and D).

## Discussion

Breast cancer remains a significant health problem worldwide and is the most common cancer diagnosed in females as it increases morbidity and mortality rates^16^. Treatment options include surgery, hormonal therapy, radiotherapy, and chemotherapy^17^. GCB remains as one of the most common chemotherapeutic agents for the treatment of breast cancer^18^. Although GCB treatment cause initial responses, its long-term success may be discontinued because of the development of drug resistance and toxicities^9,19^. Recently, the combination of natural products with chemotherapies have attracted researchers’ attention as it was found to augment the effect of standard cancer chemotherapeutic drugs and/or protect from its side effects^20–22^. TQ is among the active components of *Nigella sativa* which is commonly used for several medicinal purposes^11,23^.

Herein, we showed a strong synergism between GCB and TQ against breast adenocarcinoma (MCF-7), as well as breast ductal carcinoma (T47D) cells. It is also worth mentioning the weaker cytotoxic effect of GCB against breast cancer cells by longer exposure (72 h) might be attributed to its stability issues. GCB is unstable in serum condition and this is due to protein binding and enzyme dependent and independent degradation^24,25^. Moreover, GCB suffers from several physico-chemical stability issues in solutions^26^. Accordingly, further detailed assessment for GCB-induced influences to cell cycle, apoptosis and autophagy were carried out after treatment for 24 and 48 h. According to our observation, TQ alone showed significant but weak anti-proliferative effects in comparison to GCB. However, TQ enhanced the cytotoxic profile of GCB by 9–15 folds and 6–25 folds against MCF-7 and T47D, respectively. Several publications reported the significance of TQ alone as an anti-cancer agent in different types of cancer^27–29^. In addition, several studies including ours showed promising chemomodulatory effects of TQ to several chemotherapeutic agents against different types of cancer^15,30^. Earlier in 2014, Pandita and colleagues reported a synergistic interaction between TQ and GCB against pancreatic cancer cells. TQ down regulate Pyruvate kinase which is involved in a wide range of cancer cell metabolism^22^. Later on, Zhang and colleagues showed a chemosensetizing effect of TQ to cisplatin against colorectal cancer cells via inhibiting NF-κB signaling^31^.

In the current work, we tried to further explain the synergistic interaction between GCB and TQ in breast cancer cells from the aspect of cell cycle interference. GCB slowed down the cell cycle progression in G_0_/G_1_ and S-phases in both cell lines which was also reported by previous studies^32^. The anti-proliferative effect of GCB alone or in combination with TQ was found to be stressful enough to induce cell death observed by increased Pre-G cell population. TQ alone did not induce any significant cell cycle interference; except delayed S-phase arrest after 48 h of exposure. However, TQ was found to potentiate the killing effect of GCB increasing the Pre-G cell population in both cell lines under investigation compared to GCB treatment alone. Previous studies for the influence of TQ to cell cycle progression denoted interference with different cell cycle phases such as G_0_/G_1_ and S-phases^33,34^. TQ-induced cell cycle arrest in S-phase was also stressful to T47D cells and induced elevated Pre-G cell population. The increased Pre-G cell population might not be specific enough to determine the exact cell death mechanism. Some studies referred TQ-induced anticancer effect to its ability to induce apoptosis via TGF-family, p53, p21, c-FLIP, Bax and Bcl-2 interference^35–37^.

Furthermore, we examined apoptotic, necrotic and autophagic cell death induced by GCB, TQ and their combination. According to our observation, both TQ and GCB induced significant apoptosis in T47D by more than 4 folds after 24 h. Yet, the synergistic interaction between TQ and GCB against T47D cells could be clearly explained by the excessive increase of apoptotic cell fraction compared to single GCB or TQ treatments (2.5 fold higher apoptosis). Altered apoptosis is one of the important underlying reasons for GCB resistance among cancer cells^38,39^. Many altered pathways were suggested to be responsible for GCB-induced apoptosis resistance, such as Bcl-2^40^. It is worth mentioning that combination treatment induced significantly higher necrosis compared to GCB or TQ treatments alone which in turn might bypass apoptosis pathways altogether. In MCF-7 cells, apoptosis is not detectable due to lack of caspase-3 expression^41^. In such cases, alternative cell death pathways are mandatory. Autophagy is another suggested cell death pathway; however it possesses complicated roles and controversy in cancer cell death^42,43^. In MCF-7 cells, TQ induced significant autophagic cell death and this might be a pro-death mechanism due to defective apoptosis in this cell line^41,43^. It was reported for MCF-7 cells to undergo programmed cell death dominantly via autophagy^43^. In contrast to MCF-7, TQ did not exert any autophagic response in T47D. Furthermore, GCB and combination of GCB with TQ significantly decreased autophagic cell death. Yet, this might be explained by forced apoptosis induction in T47D cell after these treatments. In other words, autophagy is considered herein as an apoptosis escape shelter^43,44^.

Besides the synergistic interaction between GCB and TQ against breast cancer cells, we studied the influence of these treatments against breast cancer associated stem cells (CD44^+^/CD24^−^)^45^. To the best of our knowledge, this is the first study demonstrating the effect of GCB and TQ against breast cancer stem cells. According to our observations, GCB alone minimally affected CD44^+^/CD24^−^ cell clone only in MCF-7 cells. Yet, it was found that GCB treatment activates a group of developmental pathways known to be responsible for chemotherapeutic treatment resistance^9^. Interestingly, TQ significantly decreased CD44^+^/CD24^−^ cell clone in both cell lines under investigation; and TQ combination with GCB further suppressed this stem cell clone in MCF-7 cells.

In conclusion, TQ proved and is still proving to possess strong chemomodulatory potential to many chemotherapeutic agents such as GCB, against breast cancer cell lines. TQ induces cell death via apoptosis, necrosis and autophagy. In addition, TQ decreases tumor associated resistant stem cell fraction.

## Materials and Methods

### Chemicals and drugs

Thymoquinone (TQ), gemcitabine (GCB), sulpharodamine-B (SRB) were purchased from Sigma-Aldrich Chemical Co. (St. Louis, MO, USA). Media, fetal bovine serum (FBS) and other cell culture materials were purchased from Gibco^™^, Thermo Fisher Scientific (Grand Island, NY, USA).

### Cell culture

Human breast cancer cell lines, MCF-7 and T47D, were obtained from the Vaccera (Giza, Egypt). Cells were maintained in DMEM media supplemented with streptomycin (100 μg/mL); penicillin (100 units/mL) and heat-inactivated fetal bovine serum (10% v/v) in a humidified, 5% (v/v) CO_2_ atmosphere at 37 °C.

### Cytotoxicity assays

The cytotoxicity of TQ, GCB, and their combination were tested against MCF-7 and T47D cells by sulforhodamine B (SRB) assay. Exponentially growing cells were collected using 0.25% Trypsin-EDTA and seeded in 96-well plates at 1000–2000 cells/well. Cells were treated with serial concentration (0.01 to 300 µM) of TQ, GCB and their combination for 24, 48 and 72 h and subsequently fixed with trichloroacetic acid (TCA) (10% w/v) for 1 h at 4 °C. After several washings with double distilled water, cells were stained with SRB solution 0.4% (w/v) for 10 min in a dark place at room temperature and finally washed with 1% (v/v) acetic acid. After the plates became dry by overnight incubation, Tris-HCl (50 mM, pH 7.4) was used to dissolve the SRB-stained cells and color intensity was measured at 540 nm with ELISA microplate reader and calculated as percent viability of control cells (cells exposed to drug free media).

### Data analysis

The dose response curves of drugs under investigation were analyzed using E_max_ model in the following formula

1 $$ \% \,Cell\,Viability=(100-R)\times (1-\frac{{[D]}^{m}}{{K}_{d}^{m}+{[D]}^{m}})$$

where “R” is the residual unaffected fraction (the resistance fraction); “[D]” is the drug concentration used; “K_d_” is the drug concentration that produces 50% reduction of the maximum inhibition rate and m is a Hill-type coefficient. “IC_50_” is defined as the drug concentration required to reduce absorbance to 50% of the control (i.e., K_d_ = IC_50_ when R = 0 and E_max_ = 100 − R).

Combination index (CI) was calculated from the formula:

2 $$CI=\,\frac{I{C}_{50}\,of\,drug\,{(x)}_{combination}}{I{C}_{50}\,of\,drug\,{(x)}_{alone}}+\frac{I{C}_{50}\,of\,drug\,{(y)}_{combination}}{I{C}_{50}\,of\,drug\,{(y)}_{alone}}$$

The nature of drug interaction is defined as synergism if CI < 0.8; antagonism if CI > 1.2; and additive if CI ranges from 0.8–1.2.

### Analysis of Cell Cycle Distribution

To assess the effect of the TQ, GCB and their combination on cell cycle distribution, MCF-7 and T47D cells were subjected to the pre-determined IC_50_’s of test drugs or drug free media for 24 and 48 h. After treatment, cells were collected by trypsinization and washed twice with ice-cold PBS and re-suspended in 0.5 mL of PBS. Two milliliters of 60% ice-cold ethanol were added gently while vortexing and cells were incubated at 4 °C for 1 h for fixation. Upon analysis, fixed cells were washed and re-suspended in 1 mL of PBS containing 50 µg/mL RNAase A and 10 µg/mL propidium iodide (PI). After 20 min of incubation in dark at 37 °C, cells were analyzed for DNA contents using flow cytometry analysis FL2 (λ_ex/em_ 535/617 nm) signal detector (ACEA Novocyte™ flowcytometer, ACEA Biosciences Inc., San Diego, CA, USA). For each sample, 12,000 events were acquired. Cell cycle distribution was calculated using ACEA NovoExpress™ software (ACEA Biosciences Inc., San Diego, CA, USA).

### Apoptosis assay

To elucidate the method of cell death by which breast cancer cells are killed in response to treatment with GCB, TQ and their combination, apoptosis and necrosis cell populations were determined using Annexin V-FITC apoptosis detection kit (Abcam Inc., Cambridge Science Park, Cambridge, UK). Briefly, the cells were exposed to the predetermined IC_50_’s of test drugs (single or combined treatments) or drug free media (control group) for 24 h and 48 h. Cells were harvested and washed twice with PBS, and incubated in dark with 0.5 ml of Annexin V-FITC/PI solution for 30 min in a dark place at room temperature according to manufacturer protocol. After staining,cells were injected via ACEA Novocyte™ flowcytometer (ACEA Biosciences Inc., San Diego, CA, USA) and analyzed for FITC and PI fluorescent signals using FL1 and FL2 signal detector, respectively (λ_ex/em_ 488/530 nm for FITC and λ_ex/em_ 535/617 nm for PI). For each sample, 12,000 events were acquired and positive FITC and/or PI cells were quantified by quadrant analysis and calculated using ACEA NovoExpress™ software (ACEA Biosciences Inc., San Diego, CA, USA).

### Autophagy assay

To further elucidate the method of cell death by which breast cancer cells are killed in response to treatment with GCB, TQ and their combination, Autophagic cell death was quantitatively assessed using Cyto-ID Autophagy Detection Kit (Abcam Inc., Cambridge Science Park, Cambridge, UK). In brief, cells were exposed to the predetermined IC_50_’s of test compounds (single or combined treatments) for 24 h. Simultaneously, cells were exposed to 10 µM chloroquine (CQ) as a positive control (autophagy inducing agent), and drug free media (control group) for 24 h. After treatment, cells were collected and washed twice with PBS. Cells were stained with Cyto-ID Green and incubated in a dark place at 37 °C for 30 minutes according to manufacturer protocol. After staining, cells were injected via ACEA Novocyte™ flowcytometer (ACEA Biosciences Inc., San Diego, CA, USA) and analyzed for Cyto-ID differential green/orange fluorescent signals using FL1 and FL2 signal detector, respectively (λ_ex/em_ 488/530 nm for FITC and λ_ex/em_ 535/617 nm for PI). For each sample, 12,000 events were acquired and mean green fluorescent intensities (NFI) were quantified using ACEA NovoExpress™ software (ACEA Biosciences Inc., San Diego, CA, USA).

### Stem cell detection

The effects of TQ, GCB, and their combination against breast cancer associated stem cell clone (CD44^+^/CD24^−^) were assessed using flow cytometry coupled with FITC labeled anti-CD44 and APC/Cy7 labeled anti-CD24 antibodies (Abcam Inc., Cambridge Science Park, Cambridge, UK). Briefly, cells were treated for 24 h with the predetermined IC_50_’s of test compounds (single or combined treatments), and drug free media (control group). After treatment, cells were collected and washed with 10% FBS in ice cold PBS. Cells were incubated with the conjugated anti-CD44 and anti-CD24 antibodies in a dark place at room temperature. After staining, cells were washed three times with 10% FBS in ice cold PBS. Finally, cells were injected via ACEA Novocyte™ flowcytometer (ACEA Biosciences Inc., San Diego, CA, USA) and analyzed for FITC and APC/CY7 fluorescent signals using FL1 and FL2 signal detector, respectively (λ_ex/em_ 488/530 nm for FITC and λ_ex/em_ 535/617 nm for APC/CY7). For each sample, 12,000 events were acquired and positive FITC and/or APC/CY7 cells were quantified by quadrant analysis and calculated using ACEA NovoExpress™ software (ACEA Biosciences Inc., San Diego, CA, USA).

### Statistical analysis

Data are presented as mean ± SD using Prism^®^ for Windows, ver. 5.00 (GraphPad Software Inc., La Jolla, CA, USA). Analysis of variance (ANOVA) with LSD post hoc test was used for testing the significance using SPSS^®^ for windows, version 17.0.0. p < 0.05 was taken as a cut off value for significance.

## Electronic supplementary material

Supplementary Information 

## References

[1] Torre LA (2015). Global cancer statistics, 2012. CA Cancer J Clin.

[2] Montazeri A (2008). Health-related quality of life in breast cancer patients: a bibliographic review of the literature from 1974 to 2007. J. Exp. Clin. cancer Res..

[3] Prasetyanti PR, Medema JP (2017). Intra-tumor heterogeneity from a cancer stem cell perspective. Mol. Cancer.

[4] Bozorgi A, Khazaei M, Khazaei MR (2015). New Findings on Breast Cancer Stem Cells: A Review. J. Breast Cancer.

[5] Wang K (2016). Synergistic chemopreventive effects of curcumin and berberine on human breast cancer cells through induction of apoptosis and autophagic cell death. Sci. Rep..

[6] Barton-Burke M (1999). Gemcitabine: a pharmacologic and clinical overview. Cancer Nurs..

[7] Van Moorsel CJA, Peters GJ, Pinedo HM (1997). Gemcitabine: future prospects of single-agent and combination studies. Oncologist.

[8] Burstein HJ (2000). Side effects of chemotherapy. J. Clin. Oncol..

[9] Jia Y, Xie J (2015). Promising molecular mechanisms responsible for gemcitabine resistance in cancer. Genes Dis..

[10] Ko E-Y, Moon A (2015). Natural Products for Chemoprevention of Breast Cancer. J. cancer Prev..

[11] Ahmad A (2013). A review on therapeutic potential of Nigella sativa: A miracle herb. Asian Pac. J. Trop. Biomed..

[12] Dastjerdi MN, Mehdiabady EM, Iranpour FG, Bahramian H (2016). Effect of Thymoquinone on P53 Gene Expression and Consequence Apoptosis in Breast Cancer Cell Line. Int. J. Prev. Med..

[13] Pazhouhi M, Sariri R, Rabzia A, Khazaei M (2016). Thymoquinone synergistically potentiates temozolomide cytotoxicity through the inhibition of autophagy in U87MG cell line. Iran. J. Basic Med. Sci..

[14] Bayat Mokhtari R (2017). Combination therapy in combating cancer. Oncotarget.

[15] Alaufi, O. M., Noorwali, A., Zahran, F., Al-Abd, A. M. & Al-Attas, S. Cytotoxicity of thymoquinone alone or in combination with cisplatin (CDDP) against oral squamous cell carcinoma *in vitro*. *Sci*. *Rep*. **7** (2017). 10.1038/s41598-017-13357-5 PMC5640598 29030590

[16] Ghoncheh M, Pournamdar Z, Salehiniya H (2016). Incidence and Mortality and Epidemiology of Breast Cancer in the World. Asian Pac. J. Cancer Prev..

[17] Shah R, Rosso K, Nathanson SD (2014). Pathogenesis, prevention, diagnosis and treatment of breast cancer. World J. Clin. Oncol..

[18] Silvestris N (2008). Role of gemcitabine in metastatic breast cancer patients: A short review. The Breast.

[19] Dorman SN (2016). Genomic signatures for paclitaxel and gemcitabine resistance in breast cancer derived by machine learning. Mol. Oncol..

[20] Al-Abbasi, F. A. *et al*. Gingerol synergizes the cytotoxic effects of doxorubicin against liver cancer cells and protects from its vascular toxicity. *Molecules* **21** (2016). 10.3390/molecules21070886 PMC6274287 27399668

[21] Khaleel, S. A., Al-Abd, A. M., Ali, A. A. & Abdel-Naim, A. B. Didox and resveratrol sensitize colorectal cancer cells to doxorubicin via activating apoptosis and ameliorating P-glycoprotein activity. *Sci*. *Rep*. **6** (2016). 10.1038/srep36855 PMC5107943 27841296

[22] Pandita A (2014). Synergistic combination of gemcitabine and dietary molecule induces apoptosis in pancreatic cancer cells and down regulates PKM2 expression. Plos One.

[23] Khader M, Eckl PM (2014). Thymoquinone: an emerging natural drug with a wide range of medical applications. Iran. J. Basic Med. Sci..

[24] Kiew L-V, Cheong S-K, Sidik K, Chung L-Y (2010). Improved plasma stability and sustained release profile of gemcitabine via polypeptide conjugation. Int. J. Pharm..

[25] Tsume Y, Incecayir T, Song X, Hilfinger JM, Amidon GL (2014). The development of orally administrable gemcitabine prodrugs with D-enantiomer amino acids: enhanced membrane permeability and enzymatic stability. Eur. J. Pharm. Biopharm..

[26] Xu Q, Zhang V, Trissel LA (1999). Physical and chemical stability of gemcitabine hydrochloride solutions. J. Am. Pharm. Assoc..

[27] Kus G (2018). Antiproliferative and antiapoptotic effect of thymoquinone on cancer cells *in vitro*. Bratisl. Lek. Listy.

[28] Kotowski U (2017). Effect of thymoquinone on head and neck squamous cell carcinoma cells *in vitro*: Synergism with radiation. Oncol. Lett..

[29] Soltani A, Pourgheysari B, Shirzad H, Sourani Z (2017). Antiproliferative and Apoptosis-Inducing Activities of Thymoquinone in Lymphoblastic Leukemia Cell Line. Indian J. Hematol. Blood Transfus..

[30] Jafri SH (2010). Thymoquinone and cisplatin as a therapeutic combination in lung cancer: *In vitro* and *in vivo*. J. Exp. Clin. Cancer Res..

[31] Zhang L, Bai Y, Yang Y (2016). Thymoquinone chemosensitizes colon cancer cells through inhibition of NF-κB. Oncol. Lett..

[32] Miao X, Koch G, Ait-Oudhia S, Straubinger RM, Jusko WJ (2016). Pharmacodynamic Modeling of Cell Cycle Effects for Gemcitabine and Trabectedin Combinations in Pancreatic Cancer Cells. Front. Pharmacol..

[33] Wirries A, Breyer S, Quint K, Schobert R, Ocker M (2010). Thymoquinone hydrazone derivatives cause cell cycle arrest in p53-competent colorectal cancer cells. Exp. Ther. Med..

[34] Raghunandhakumar S (2013). Thymoquinone inhibits cell proliferation through regulation of G1/S phase cell cycle transition in N-nitrosodiethylamine-induced experimental rat hepatocellular carcinoma. Toxicol. Lett..

[35] Park EJ, Chauhan AK, Min K-J, Park DC, Kwon TK (2016). Thymoquinone induces apoptosis through downregulation of c-FLIP and Bcl-2 in renal carcinoma Caki cells. Oncol. Rep..

[36] Liu X (2017). The effect of thymoquinone on apoptosis of SK-OV-3 ovarian cancer cell by regulation of Bcl-2 and Bax. Int. J. Gynecol. Cancer.

[37] Diab-Assaf M (2018). Inhibition of Proliferation and Induction of Apoptosis by Thymoquinone via Modulation of TGF Family, p53, p21 and Bcl-2α in Leukemic Cells. Anti-Cancer Agents Med. Chem. (Formerly Curr. Med. Chem. Agents).

[38] Adamska, A. *et al*. Molecular and cellular mechanisms of chemoresistance in pancreatic cancer. *Adv*. *Biol*. *Regul* (2017). 10.1016/j.jbior.2017.11.007 29221990

[39] Amrutkar M, Gladhaug IP (2017). Pancreatic cancer chemoresistance to gemcitabine. Cancers (Basel)..

[40] Bold RJ, Chandra J, McConkey DJ (1999). Gemcitabine-induced programmed cell death (apoptosis) of human pancreatic carcinoma is determined by Bcl-2 content. Ann. Surg. Oncol..

[41] Jänicke RU (2009). MCF-7 breast carcinoma cells do not express caspase-3. Breast Cancer Res. Treat..

[42] Towers CG, Thorburn A (2016). Therapeutic targeting of autophagy. EBioMedicine.

[43] Hippert MM, O’Toole PS, Thorburn A (2006). Autophagy in cancer: good, bad, or both?. Cancer Res..

[44] Jin S, White E (2007). Role of autophagy in cancer: management of metabolic stress. Autophagy.

[45] Horimoto Y (2016). Combination of Cancer Stem Cell Markers CD44 and CD24 Is Superior to ALDH1 as a Prognostic Indicator in Breast Cancer Patients with Distant Metastases. Plos One.

