# Supplementary material for: Thymoquinone synergizes gemcitabine anti-breast cancer activity via modulating its apoptotic and autophagic activities
Source: Sci Rep. 2018 Aug 3;8:11674. doi: 10.1038/s41598-018-30046-z (PMC6076303; doi:10.1038/s41598-018-30046-z)
Supplement: Supplementary file 1 — Supplementary Information [file 41598_2018_30046_MOESM1_ESM.pdf]

Thymoquinone synergizes gemcitabine anti-breast cancer activity via modulating its apoptotic and autophagic activities

Hanan A. Bashmail[1], Aliaa A. Alamoudi[1,2], Abdulwahab Noorwali[1,2], Gehan A. Hegazy[1,3], Ghada AJabnoor[1], Hani Choudhry[4], Ahmed M. Al-Abd [5,6,7,*]

[1]Departement of Clinical Biochemistry, Faculty of Medicine, King Abdulaziz University, Jeddah, Saudi Arabia

[2]Stem Cell Research Unit, King Fahad Medical Research Center, Jeddah, Saudi Arabia

[3]Department of Hormones, Medical Division, National Research Centre, Giza, Egypt

[4]Department of Biochemistry, Faculty of Science, King Abdulaziz University, Jeddah, Saudi Arabia

[5]Department of Pharmacology, Medical Division, National Research Centre, Giza, Egypt

[6]Biomedical Research Section, Nawah Scientific, Mokkatam, Cairo, Egypt

[7]Depaertment of Pharmaceutical Sciences, College of Pharmacy, Gulf Medical University, Ajman, United Arab Emirates

[*]Corresponding authors:

Ahmed M. Al-Abd, Department of Pharmacology, Medical Division, National Research Centre, Giza, Egypt

Tel: +966-(0)2-640-0000/Ext 21125

Fax: +966-(0)2-695-1696

E-mail: ahmedmalabd@pharma.asu.edu.eg

Supp. Table 1. The effect of TQ, GCB and their combination on cell cycle distribution of MCF-7 cell line after 24 h treatment

Phase	Control	TQ	GCB	GCB+TQ
**Go / G1**	38.1±3.0%	63.9±0.7%	67.7±1.2%	62.1±1.0%
**S**	44.5±2.5%	44.4±0.3%	24.6±1.5%	23.3±0.7%
**G2 / M**	17.5±2.3%	18.7±0.3%	7.6±0.9%	14.5±1.0%
**Pre-G**	4.7±1.5%	3.3±0.2%	8.8±0.6%	7.2±1.2%

Supp. Table 2. The effect of TQ, GCB and their combination on cell cycle distribution of MCF-7 cell line after 48 h treatment

Phase	Control	TQ	GCB	GCB+TQ
**Go / G1**	46.1±3.9%	43.2±6.9%	26.4±3.6%	22.4±2.2%
**S**	38.0±1.9%	40.4±2.9%	50.4±3.2%	48.2±4.7%
**G2 / M**	15.9±2.0%	16.4±3.9%	23.2±3.0%	29.4±3.0%
**Pre-G**	2.5±0.6%	2.5±0.6%	23.1±3.4%	49.3±4.2%

Supp. Table 3. The effect of TQ, GCB and their combination on cell cycle distribution of T47D cell line after 24 h treatment

Phase	Control	TQ	GCB	GCB+TQ
**Go / G1**	53.2±0.6%	47.7±3.5%	60.5±1.8%	58.9±3.9%
**S**	27.5±1.9%	32.4±4.1%	27.3±1.7%	33.1±4.6%
**G2 / M**	19.4±1.7%	19.9±2.2%	12.2±0.6%	8.0±1.5%
**Pre-G**	8.5±0.3%	10.8±0.2%	28.9±0.9%	57.1±4.4%

Supp. Table 4. The effect of TQ, GCB and their combination on cell cycle distribution of T47D cell line after 24 h treatment

Phase	Control	TQ	GCB	GCB+TQ
**Go / G1**	54.3±2.7%	52.7±1.4%	36.5±3.4%	32.1±3.3%
**S**	29.1±1.7%	38.4±0.2%	44.3±2.5%	49.9±1.3%
**G2 / M**	16.6±1.0%	8.8±1.2%	19.2±1.7%	18.1±3.6%
**Pre-G**	12.6±1.4%	67.6±5.2%	15.1±1.1%	64.5±1.0%
